# Gas2l3, a Novel Constriction Site-Associated Protein Whose Regulation Is Mediated by the APC/C^Cdh1^ Complex

**DOI:** 10.1371/journal.pone.0057532

**Published:** 2013-02-28

**Authors:** Tal Pe’er, Roxane Lahmi, Yaara Sharaby, Evelin Chorni, Meirav Noach, Manuela Vecsler, Eitan Zlotorynski, Hanno Steen, Judith A. Steen, Amit Tzur

**Affiliations:** 1 The Mina and Everard Goodman Faculty of Life Sciences, Bar-Ilan University, Ramat-Gan, Israel; 2 Institute of Nanotechnology and Advanced Materials, Bar-Ilan University, Ramat-Gan, Israel; 3 Proteomics Center, Boston Children’s Hospital, Boston, Massachusetts, United States of America; 4 Department of Pathology, Boston Children’s Hospital, Boston, Massachusetts, United States of America; 5 Department of Pathology, Harvard Medical School, Boston, Massachusetts, United States of America; 6 Department of Neurobiology, Harvard Medical School and F. M. Kirby Neurobiology Center, Boston Children’s Hospital, Boston, Massachusetts, United States of America; University of Leicester, United Kingdom

## Abstract

Growth arrest-specific 2-like protein 3 (Gas2l3) was recently identified as an Actin/Tubulin cross-linker protein that regulates cytokinesis. Using cell-free systems from both frog eggs and human cells, we show that the Gas2l3 protein is targeted for ubiquitin-mediated proteolysis by the APC/C^Cdh1^ complex, but not by the APC/C^Cdc20^ complex, and is phosphorylated by Cdk1 in mitosis. Moreover, late in cytokinesis, Gas2l3 is exclusively localized to the constriction sites, which are the narrowest parts of the intercellular bridge connecting the two daughter cells. Overexpression of Gas2l3 specifically interferes with cell abscission, which is the final stage of cell division, when the cutting of the intercellular bridge at the constriction sites occurs. We therefore suggest that Gas2l3 is part of the cellular mechanism that terminates cell division.

## Introduction

Dividing cells ensure the symmetric distribution of their genetic material by multiple modes of regulation. A series of phosphorylation events, as well as strictly regulated proteolysis, mediated mainly by the Anaphase-Promoting Complex/Cyclosome (APC/C) E3 ubiquitin ligase, coordinate the spindle assembly checkpoint removal and sister-chromatid separation, and the metaphase-to-anaphase transition [1], [2]. In parallel, cytokinesis, which starts with the formation of the cleavage furrow, halves the cell’s cytoplasm and eventually two daughter cells are formed [3]. APC/C in complex with Cdh1 targets key cytokinesis regulators, such as the mitotic kinases Plk1 and Aurora B, and Anillin, for proteasome-dependent degradation in late telophase and throughout the G1 phase of the cell cycle [4], [5]. This proteolysis has been shown to be important for progression through cytokinesis and mitotic exit [4], [6].

The final step of the cell cycle is cell abscission; that is, the cutting of the thin microtubule and membrane bridge connecting the two daughter cells at specific locations on both sides of the midbody, called the constriction sites (also known as the “abscission zones”) [7]. At these sites, the microtubule bundles are at their narrowest [8], [9], [10]. The molecular mechanisms underlying cell abscission and the way in which abscission is coordinated with the cell cycle, are still mostly unclear; nevertheless, it has been shown recently that components of the ESCRT membrane transport system, in particular the ESCRT-III complex [8], [9] and the microtubule-severing machinery [7], [11], are recruited to the constriction sites to trigger the microtubule breakage and membrane scission that end the cell division process. In addition, recent findings suggest that orderly abscission is, at least in part, mediated by the Plk1-regulated recruitment of abscission factors [6].

Partitioning the cell content requires components of the Actin filament network, in particular those of the contractile ring, as well as components of the microtubule network and of the cell membrane [3]. Coordinating these components together enables the synchronization of chromosome segregation with cleavage furrow ingression and the actual act of cell division, but it is also essential for a variety of fundamental cellular activities, such as cell polarization and migration. A series of cross-linker proteins, in particular spectraplakins, can directly interact with Actin filaments and microtubules, as well as with intermediate filaments, and integrate them into one cytoskeleton network that can also communicate with other cellular structures, such as membranes [12].

Growth arrest-specific 2-like (Gas2l) proteins comprise a subfamily of spectraplakin-like cross-linking factors that bind Actin filaments and microtubules through their Calponin homology (CH) and GAS2-related (GAR) domains, and possibly through other binding domains [13], [14], [15]. The family includes three proteins: Gas2l1, Gas2l2, and Gas2l3, all named after their GAR signature motifs. The expression of Gas2 itself was identified to be specifically elevated during cell growth arrest [16], and has been shown to inhibit cell division and activate apoptosis in vertebrate embryos [15], [17], [18].

It was recently reported that GAS2L3 transcription is cell cycle-regulated via the DREAM/E2F protein complex. Furthermore, the results of RNAi experiments have suggested a role for Gas2l3 in cytokinesis [19]. Here we show that Gas2l3 is a novel constriction site-associated protein and an APC/C^Cdh1^ target whose overexpression interferes with cell abscission.

## Results

### Gas2l3 is a Cell Cycle-regulated Protein and a Novel Substrate of the APC/C^Cdh1^ Complex

**Figure 1:**
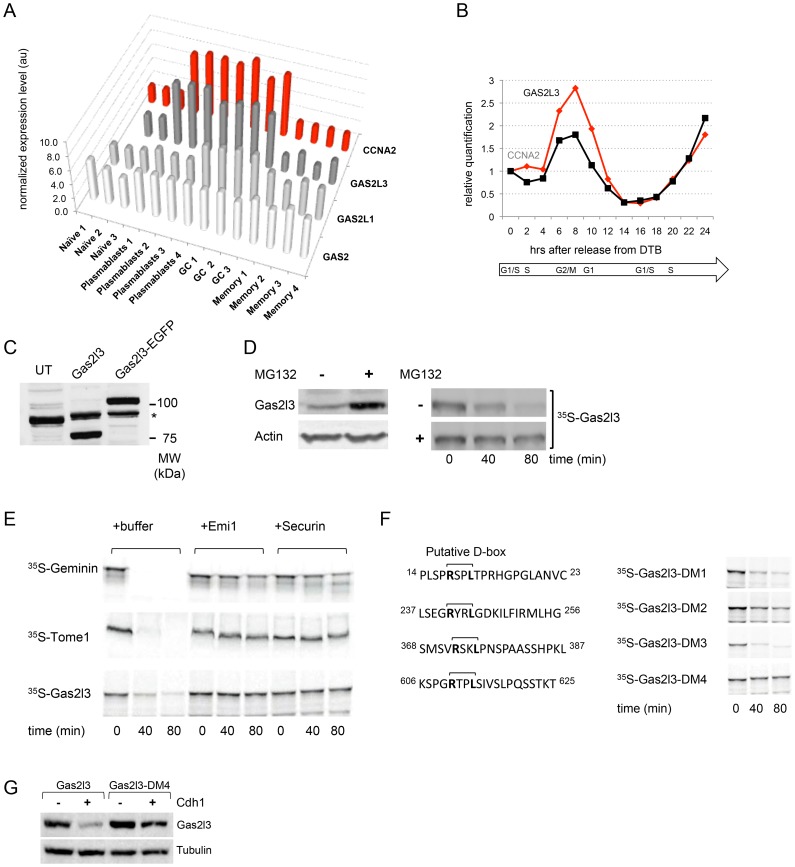
Gas2l3 is a cell cycle-regulated protein whose degradation is mediated by the APC/C. (A) Normalized expression levels of the mouse genes GAS2, GAS2L1, GAS2L3, and CCNA2 in naïve, plasmablast, germinal center (GC), and memory-B cells. Raw data were taken from the GEO database GSE4142. (B) Real-time PCR analysis of human GAS2L3 and CCNA2 expression throughout the cell cycle of HeLa S3 (S3) cells, normalized to ACTB RNA levels at time point 0. Cells were double-thymidine blocked, released, and harvested for RNA extraction every 2 hrs for 24 hrs. Cell cycle progression of the synchronous populations (measured by propidium-iodide (PI) staining followed by FACS analysis) is depicted under the plot. (C) HEK293 (293) cells overexpressing human Gas2l3 or Gas2l3-EGFP were harvested for Western blot analysis with custom-made polyclonal hGas2l3 antibodies (serum). A cross-reactive band is noted (asterisk) in the transfected and untransfected (UT) cells. Forty µg extracts made from transfected cells and 120 µg extracts made from untransfected cells were assayed. (D) Left: 293 cells were transfected with the Gas2l3 expression vector. After 24 hrs, cells were treated with MG132 for 5 hrs and subjected to Western blotting with Gas2l3 (serum) and Actin antibodies (loading control). Right: Human Gas2l3 was expressed in rabbit reticulocytes supplemented with radiolabeled (^35^S) methionine. *In vitro* transcribed/translated (IVT) Gas2l3 product was incubated in G1 extracts of S3 cells in the presence of MG132 or DMSO (control). Time-dependent degradation was assayed by SDS-PAGE and autoradiography. (E) ^35^S-labeled hGeminin, hTome-1, and hGas2l3 IVT products were incubated in G1-phase S3 cell extracts supplemented with either buffer, the C-terminus of hEmi1, or hSecurin. Time-dependent degradation was assayed by SDS-PAGE and autoradiography. (F) Left: The putative D-boxes of human Gas2l3 are depicted. Right: Arg at position 1 and Leu at position 4 of each putative D-box were substituted with Gly and Val, respectively. The degradation of the four D-box mutants (DM1–DM4) was assayed as described in (D). (G) 293 cells were cotransfected with Cdh1 (+) or empty vector (-), and with either Gas2l3 or its mutant derivate Gas2l3-DM4, at a 4∶1 ratio, respectively. After 30 hrs cells were harvested for Western blotting with anti-hGas2l3 (serum) and anti-Tubulin (control).

We found the gene encoding for growth arrest-specific 2-like protein 3 (Gas2l3) to be a putative cell cycle regulator *in vivo* using gene expression analysis of proliferating *vs.* resting murine B cells [20]. Gas2l3 belongs to the Gas2 subfamily of microtubule/Actin cross-linking factor proteins [13], [14], [15]. Of the Gas2 family, the GAS2, GAS2L1, and GAS2L3 genes were analyzed in the GSE4142 dataset [20] and out of the three, only GAS2L3, much like the mitotic cyclin A2 (CCNA2), is highly expressed in the two proliferating B-cell types (germinal center [GC] and plasmablasts) compared to the two resting B-cell types (naïve and memory). In comparison, GAS2L1 is highly expressed only in GC cells, and the expression of GAS2 is roughly equal in the four B-cell types (Figure 1A). Expression analysis of GAS2L3 in synchronous HeLa S3 (S3) cells has also shown that this gene is periodically expressed in proliferating cells in a manner similar to that of the mitotic Cyclin A2 (Figure 1B).

The molecular weight prediction (by sequence) of human Gas2l3 is 75.2 kDa. We were unable to detect the endogenous Gas2l3 by Western blotting or immunoprecipitation in numerous human cell lines using commercial antibodies. We therefore generated a polyclonal rabbit hGas2l3 antibody and tested its specificity in HEK293T (293) cells overexpressing human Gas2l3 or its EGFP-tagged version. With these antibodies, we specifically detected exogenously expressed Gas2l3 as well as the EGFP-tagged form of the protein (with a calculated molecular weight of 104.5 kDa) (Figure 1C). Nevertheless, despite our efforts, we were unable to conclusively detect by Western blotting the endogenous protein in 293 cells (Figure 1C) or in other human cell lines using our antibody.

Cell cycle proteins such as the mitotic cyclins are often tightly regulated by the ubiquitin-proteasome system, which times their destruction at specific points along the cell cycle. Treating 293 cells overexpressing Gas2l3 with the proteasome inhibitor MG132 resulted in a significant accumulation of the protein (Figure 1D, left). In order to test whether proteasome-dependent degradation of Gas2l3 takes place in G1 as it does for the mitotic cyclins, radiolabeled (^35^S) *in vitro* translated/transcribed (IVT) Gas2l3 product was incubated in cell extracts made from S3 cells synchronized in G1 [21]. Gas2l3 was almost undetectable after 80 min of incubation in control extracts, but remained stable in extracts supplemented with MG132 (Figure 1D, right), indicating that Gas2l3 undergoes proteasome-dependent degradation in G1.

We next tested whether the degradation of Gas2l3 in G1 extracts is mediated by the APC/C, which targets the mitotic cyclins and numerous other key cell cycle proteins for destruction [1]. To this end, a radiolabeled Gas2l3 IVT product was incubated in G1-phase cell extracts supplemented with either the C-terminal region of the APC/C specific inhibitor Emi1 or with an excess of Securin, a specific competitive inhibitor, both of which block APC/C-dependent degradation [21], [22]. As positive controls, we showed that radiolabeled Geminin and Tome-1, both known substrates of the APC/C [23], [24], are effectively degraded in these extracts but not in the presence of Emi1 or Securin. Results shown in Figure 1E demonstrate that Gas2l3 is degraded in G1 extracts only in the absence of Emi1 or Securin, indicating that Gas2l3 is a target of the APC/C.

APC/C substrates typically carry one or both of two recognition motifs known as the destruction box (D-box) (consensus sequence: RXXL) and the KEN-box. Gas2l3 carries four RXXL motifs, and we have generated four mutant derivates of Gas2l3 (D-box Mutant [DM] 1–4), in which the Arg and Leu at positions 1 and 4 of each of the four putative D-box sequences were substituted with Gly and Val, respectively (Figure 1F, left). Mutations at Arg610 and Leu613 (the Gas2l3-DM4 derivate) completely blocked Gas2l3 degradation in G1 extracts, while mutations at all other sites had either no (Gas2l3-DM1 and Gas2l3-DM3) or a limited (Gas2l3-DM2) effect (Figure 1F, right). We next tested the stability of Gas2l3 and Gas2l3-DM4 in 293 cells overexpressing Cdh1, which mediates the ubiquitination of nearly all known APC/C substrates [1]. Western blot analysis with anti-hGas2l3 rabbit serum revealed that exogenously expressed Gas2l3, but not Gas2l3-DM4, is highly sensitive to elevated levels of Cdh1 in cells (Figure 1G). We can therefore conclude that Gas2l3 is a *bona fide* D-box–dependent APC/C substrate.

The degradation of D-box–containing proteins is mediated either by Cdc20 (at anaphase and early telophase) and Cdh1 (at late telophase and throughout G1), or exclusively by Cdh1. This specific timing of protein degradation is a key regulatory mechanism that ensures the correct progression of cells through mitosis and G1 [1], [25]. We used the *Xenopus laevis* egg-extract system to differentiate which of the APC/C complexes drives Gas2l3 degradation: APC/C^Cdc20^ is highly active in egg extracts driven into mitosis by an excess of non-degradable Cyclin B1 [26]; meanwhile, APC/C^Cdh1^ activity, not being present in pre-mid-blastula transition (MBT) embryos, can be restored in interphase egg extracts by adding recombinant Cdh1 [24], [27].

**Figure 2:**
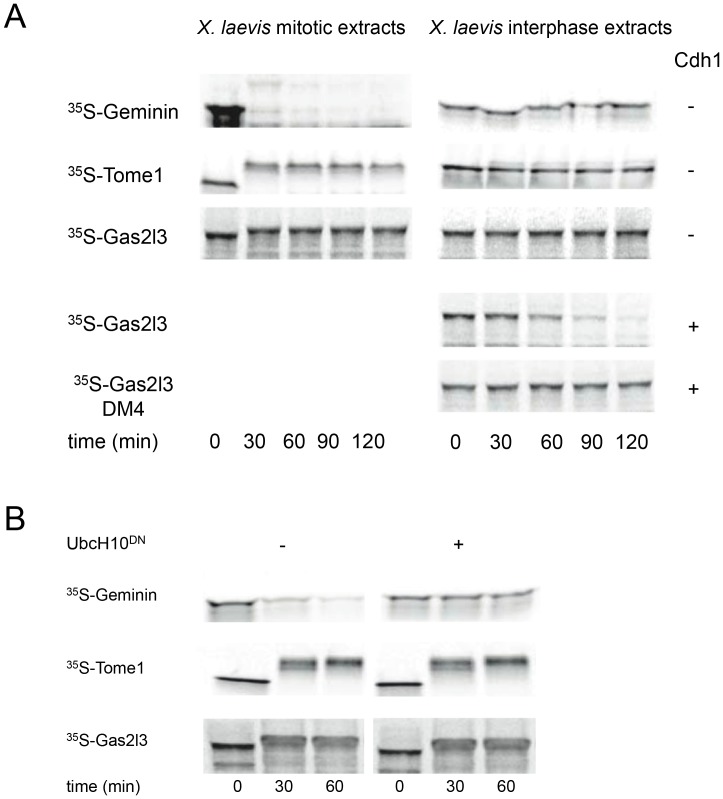
Gas2l3 degradation is mediated by APC/C^Cdh1^, but not by APC/C^Cdc20^. (A) Incubation of IVT-generated Tome-1, Geminin, Gas2l3, and Gas2l3-DM4 in *X. laevis* interphase egg extracts (right) supplemented with recombinant Cdh1 (+) or buffer (-), or in extracts driven into mitosis (left) by preincubation with recombinant non-degradable Cyclin B1 protein [Δ90 (23)]. (B) APC/C^Cdc20^-active extracts were made from 293 cells arrested in late mitosis by overexpression of His-tagged non-degradable hCyclin B1. Degradation of IVT-generated positive (Geminin) and negative (Tome-1) controls and of Gas2l3 was assayed in extracts supplemented with mock (-) or dominant negative UbcH10 (Ubch10^DN^). Time-dependent degradation was assayed by SDS-PAGE and autoradiography.

The degradation of Gas2l3 was assayed in both mitotic and interphase frog egg extracts. Geminin, an APC/C^Cdc20^ and APC/C^Cdh1^ substrate, and Tome-1, an APC/C^Cdh1^ substrate, were used as controls for the extracts’ activities and specificities. Geminin disappeared quickly in mitotic extracts unlike Tome-1, which remained stable and shifted to a high-mobility form, as previously reported (Figure 2A, left panels) [24]. Geminin is not degraded nor is Tome-1 modified in interphase extracts, thus excluding the possibility of contamination by mitotic APC/C^Cdc20^-active extracts (Figure 2A, right panels). Gas2l3 was stable in mitotic extracts (Figure 2A, left panels), but degraded in a D-box–dependent manner in interphase extracts supplemented with recombinant Cdh1 (Figure 2A, right panels).

**Figure 3:**
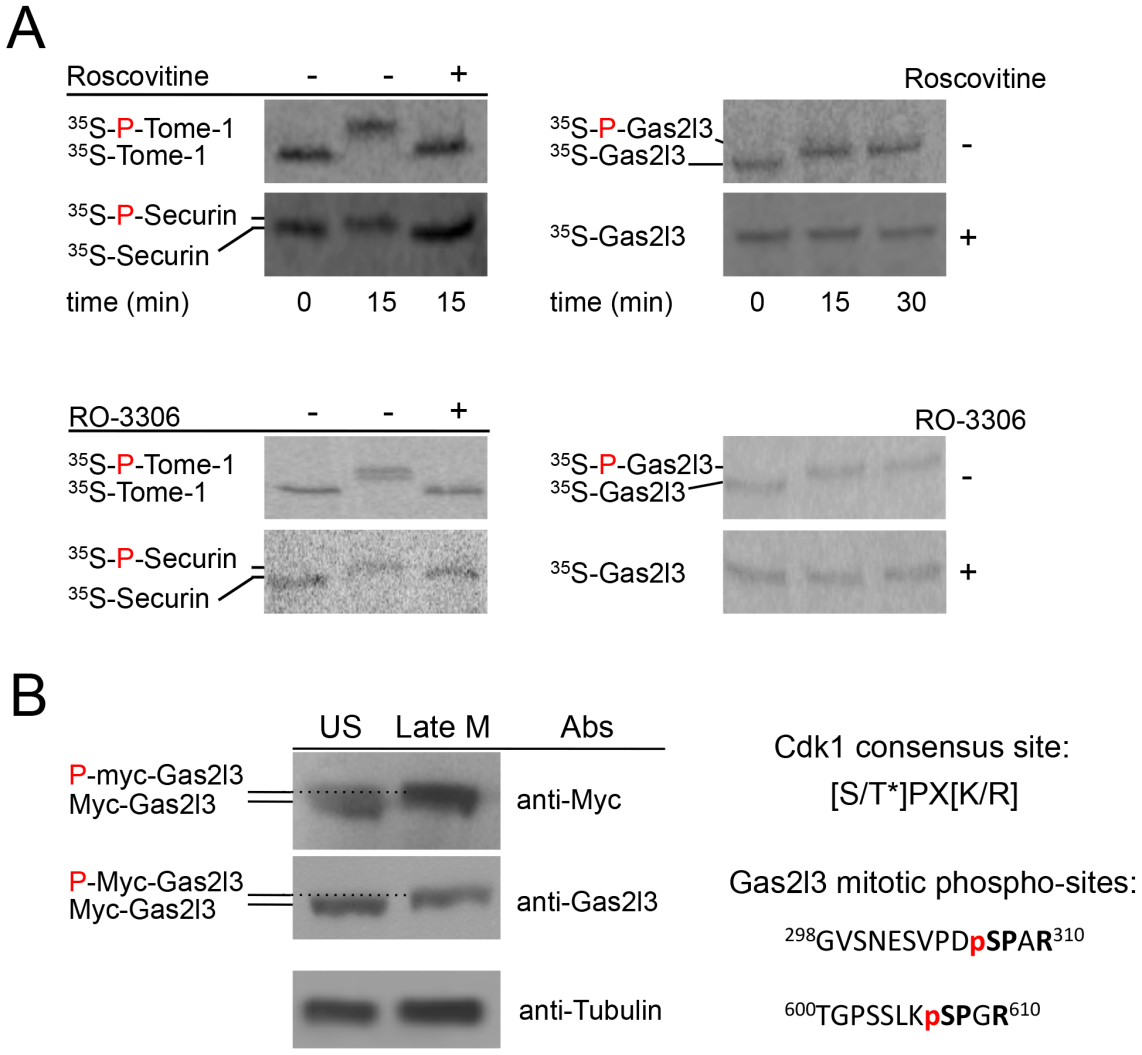
Gas2l3 is a Cdk1 target. (A) The phosphorylation of Securin, Tome-1 (positive controls), and Gas2l3 was assayed in 293 late mitotic extracts [see (B) for details] in the presence of the Cdk1 inhibitors RO-3306 or roscovitine (+), or DMSO (-). Because Securin is naturally degraded in this extract system, Securin phosphorylation was assayed in the presence of MG132. (B) 293 cells were cotransfected with Myc-tagged Gas2l3 and either non-degradable Cyclin B1 or empty vector to generate asynchronous (US) and late mitotic (late M) cell populations overexpressing Gas2l3. The cells were harvested after 24 hrs for Western blotting (with the depicted antibodies) and analysis by liquid chromatography-mass spectrometry (LC-MS/MS). The samples were separated by SDS-PAGE and the area of interest, as determined by Western blotting, was excised for in-gel digestion following established protocols. The resulting peptide mixture was analyzed using a Q Exactive mass spectrometer (Thermo Scientific) equipped with a nanoflow UPLC system (Eksigent). The LC-MS data were searched against a human protein sequence database (Uniprot/Swissprot, canonical and isoform sequence data) using ProteinPilot (v4.5, AB Sciex), allowing for biological modifications/phosphorylation emphasis. Only peptide-spectrum matches above a 1% FDR cut-off were considered for further analysis. The amino acid sequence of two phosphopeptides is depicted.

Geminin, Tome-1, and Gas2l3 degradation was also examined in somatic cell extracts with high APC/C^Cdc20^ activity made from 293 cells arrested in late mitosis by the overexpression of D-box mutant full-length Cyclin B1 [25]. This extract system, described here for the first time, can be referred to as the human equivalent of the *X. laevis* mitotic extracts, in which APC/C^Cdc20^ is highly active (for further details, see the Materials and Methods section). Geminin is quickly degraded in this system but is stabilized in the presence of Ubch10^DN^ – the dominant negative derivate of the E2 enzyme UbcH10– thus excluding the presence of any non-specific proteolytic activity in these extracts (Figure 2B). In contrast, Tome-1 remains stable irrespective of the presence of UbcH10^DN^ (Figure 2B), and shifts to the high-mobility form, as in *X. laevis* mitotic extracts (Figure 2A). Like Tome-1, Gas2l3 is stable in late mitosis extracts (Figure 2B) but is degraded in G1-phase cell extracts containing the active APC/C^Cdh1^ complex (Figure 1E). Additionally, the high-mobility forms of Gas2l3, as well as those of the two known Cdk1 targets Tome-1 and Securin [24], [28], are undetectable in the presence of the Cdk1 inhibitors RO-3306 and roscovitine (Figure 3A), suggesting that Gas2l3 is a target of Cdk1. Mass-spec analysis of gel-extracted proteins from 293 cells overexpressing Myc-tagged Gas2l3 and either non-degradable Cyclin B1 (late mitosis [M]) or empty vector (unsynchronized [US]), revealed two Cdk1 sites in Gas2l3. Moreover, these two peptides were found to be phosphorylated only in the late mitotic extracts (Figure 3B). Taken together, these results show that Gas2l3 is targeted for degradation by the APC/C^Cdh1^ complex, but not by the APC/C^Cdc20^ complex, and is a Cdk1 substrate.

### Gas2l3 is a Constriction Site-associated Protein

During interphase, Gas2l3 colocalizes with both microtubules and Actin filaments, as predicted by its CH and GAR domains and recently confirmed [13]. As cells progress through telophase, Gas2l3 becomes exclusively associated with the midzone microtubules, and later with the midbody [19].

**Figure 4:**
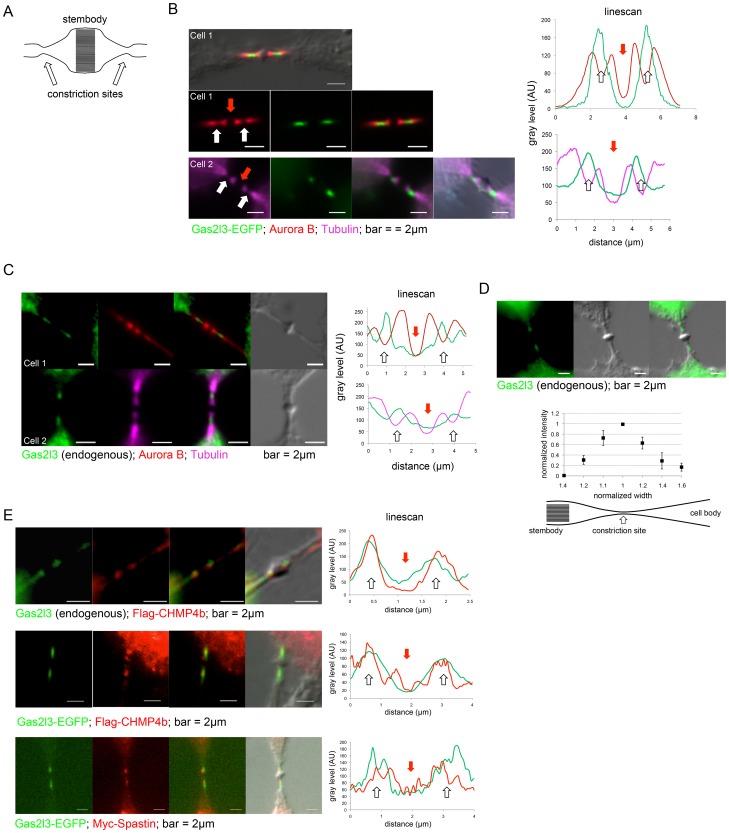
Gas2l3 is localized to the constriction sites. (A) Schematic representation of the midbody. (B) HeLa cells expressing EGFP-tagged Gas2l3 were fixed, immunolabeled with either anti-Aurora B or anti-Tubulin, and stained with DAPI. The localization of proteins to the midbody was determined using linescans (stembody and constriction sites are labeled by red and white arrows, respectively). (C) HeLa cells were fixed and immunolabeled with anti-hGas2l3 (serum), and with either anti-Aurora B or anti-Tubulin. Linescans for cells 1 and 2 are plotted. (D) HeLa cells were fixed and immunolabeled with anti-hGas2l3 (serum). The immunofluorescent intensity of the endogenous Gas2l3 at six points across one side of the midbody were measured relative to the width of the intercellular microtubule bridge at these locations (see average and standard error values for *N* = 5 at the plot, and the scheme underneath). Width and intensity values were normalized to the intercellular bridge width and Gas2l3 intensity at the constriction sites. (E) HeLa cells expressing FLAG-tagged human CHMP4b (top panels), Gas2l3-EGFP and FLAG-CHMP4b (mid panels), or Gas2l3-EGFP and Myc-tagged human Spastin (bottom panels), were fixed and immunolabeled with anti-hGas2l3 (serum [top panels]), anti-FLAG (mid panels), or anti-Myc (bottom panels). Linescans are depicted. We used a Zeiss AxioImager and 100X oil lens for imaging, and ImageJ software for image analysis.

The final stage of cytokinesis is cell abscission – the cutting of the membrane bridge connecting the two daughter cells from both sides of the stembody [3]. The constriction sites (schematically depicted in Figure 4A) are present at the narrowest part of the microtubule bridge (for a brief explanation, see Ref [7]), and can also be inferred by the less intense immunofluorescence (IF) signal of Tubulin, which most likely is the result of the dense (and thus, less accessible for IF labeling) microtubules at these sites [7], [8], [9], [10]. In addition, the recruitment of the ESCRT-III complex, in particular CHMP4b and Spastin, to the constriction sites, is considered a prerequisite for cell abscission [7], [8], [9].

EGFP-tagged Gas2l3 (Figure 4B), as well as the endogenous protein (Figure 4C), are flanked by Aurora B at the midbody (see Figures S1 and S2 for supporting information regarding Gas2l3 localization and antibody specificity). Importantly, the Gas2l3 signal peaks exactly at the constriction sites, where both the Tubulin and the Aurora B fluorescent signals are less intense (see linescans in Figures 4B and 4C) and the microtubule bridge is at its narrowest point (Figure 4D). Moreover, Gas2l3 colocalizes with both CHMP4b and Spastin at the constriction sites (Figure 4E). Endogenous Gas2l3 and both N- and C-terminal EGFP-tagged Gas2l3 are indistinguishably localized to the constriction sites (Figures 4 and S3A). Furthermore, the D-box has no effect on Gas2l3 localization to the constriction sites (Figure S3B) or any other site throughout the cell cycle (data not shown). We therefore conclude that Gas2l3 is a new member of the very small group of constriction site-associated proteins.

### Overexpression of Gas2l3 Interferes with Cell Abscission

Gas2l3 degradation requires the APC/C^Cdh1^ and, thus, is unlikely to regulate the metaphase-to-anaphase transition because Cdh1 mediates protein degradation only after cytokinesis has commenced [1]. It is therefore easy to speculate that Gas2l3 is required to coordinate cytokinesis, as was recently suggested based on RNAi experiments [19], but must be degraded as part of a mechanism that terminates cell division, as was suggested for other cytokinesis regulators that are targeted for destruction by the APC/C^Cdh1^ (*e.g.,* Plk1 and Aurora B [4], [6]).

**Figure 5:**
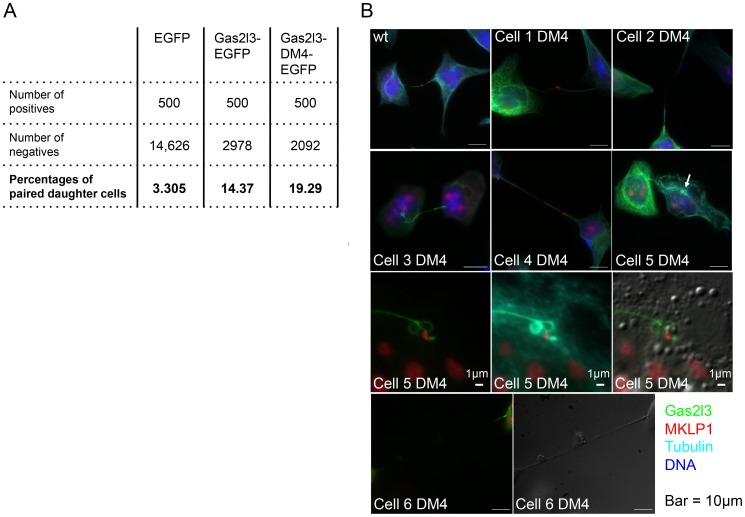
High levels of Gas2l3 interfere with cell abscission. (A) HeLa cells were transfected with vectors expressing Gas2l3-EGFP, Gas2l3-DM4-EGFP, or EGFP (control). Thirty-two hrs post-transfection, cells were fixed and immunolabeled, as described in Figure 4C. The slides were scanned using a Zeiss AxioImager upright microscope and 100X oil lens for imaging. Transfected (green) cells were counted in order to detect 500 positive daughter cell pairs connected by a midbody (positives). The proportion of attached daughter cells equals 500/*N*, where *N* = the number of transfected cells (*N* - 500 =  the number of negatives). (B) HeLa cells were transfected with vectors expressing EGFP-tagged Gas2l3 (wt) or Gas2l3-DM4. Thirty-two hours post-transfection, cells were fixed, immunolabeled with anti-MKLP1 (stembody marker) and anti-Tubulin, and stained with DAPI. We used a Zeiss AxioImager Z1 upright microscope and 100X oil lens for imaging.

In a population of cells growing exponentially, the proportion of daughter cells still attached by a midbody is expected to be low due to the relatively short duration of cytokinesis and cell division with respect to the total length of the mammalian cell cycle. In fixed HeLa cells, we measured this proportion to be 3.305% in cells transfected with EGFP as control (Figure 5A). This proportion increased over fourfold in cells overexpressing EGFP-tagged Gas2l3. Moreover, this notable phenotype was even more prevalent for the D-box mutant Gas2l3, where nearly 20% of the transfected cells were attached by an intercellular bridge (detected by the midbody marker MKLP1 [10]). This is equivalent to >6 times the frequency found in the EGFP-transfected cells, and is significantly more frequent (by 34%) in comparison to cells expressing the w.t. Gas2l3-EGFP (*p*-value [Gas2l3 w.t. *vs.* DM4] = 1.9533×10^−5^, calculated by the Fisher’s exact test) (Figure 5A). Interestingly, nearly all daughter-cell pairs overexpressing Gas2l3 were in interphase (evident by their decondensed chromatin, their shape, and the presence of nuclei), fully adherent, and although they were separated from each other – some by a large distance of over 50 microns (*e.g.,* cell 6 DM4 in Figure 5B) – they were still attached by a thin, long, and often abnormally structured microtubule bridge. For example, in cell 5 DM4 (Figure 5B), a midbody remnant, marked by the midbody ring protein MKLP1, is absorbed in one of the two daughter cells (see the white arrow and the matching magnified images), yet a thin and aberrant Tubulin bridge connecting these cells is present. These results suggest that high levels of Gas2l3 appear to permit cytokinesis but prevent cell abscission.

## Discussion

### Gas2l3 is a Cell Cycle-regulated Protein

The expression pattern of Gas2l3 in proliferating cells *in vivo* (Figure 1A) and in culture cells (Figure 1B, and [19]) resembles that of the mitotic cyclins. At the protein level, Gas2l3 interacts with the cell cycle by at least three modes. The first is proteasome-dependent protein degradation: Gas2l3 is a target of the APC/C^Cdh1^ complex and is degraded by the proteasome in a D-box–dependent manner (Figures 1 and 2). This regulation ensures that Gas2l3 levels will drop late in mitosis, when cytokinesis is in an advanced state, and will remain low until the G1/S transition. Recent RNAi experiments suggest that Gas2l3 is dispensable for mitotic-spindle formation and for metaphase and anaphase, but is required for telophase and cytokinesis [19]. The need for Gas2l3 in late mitosis is in perfect agreement with its resistance to APC/C^Cdc20^-dependent degradation (Figure 2). This also suggests that Gas2l3 degradation is unlikely to regulate sister-chromatid separation and metaphase-to-anaphase transition.

The second mode is phosphorylation: Gas2l3 is mobility-shifted in mitotic extracts by Cdk1-mediated phosphorylation (Figure 3). It is easy to speculate that this phosphorylation, perhaps in concert with the Plk1 and Aurora kinases, is an additional mode of regulation of Gas2l3 function and localization, as shown for other cytokinesis regulators. We detect Gas2l3 by SDS-PAGE slightly higher than the 75 kDa marker (Figure 1C), in agreement with the protein’s predicted molecular weight (75.2 kDa). In their recent publication, Wolter *et al.* conclusively detected Gas2l3 at 95 kDa [19]. Our findings indicate that Gas2l3 can be shifted to a higher mobility form by phosphorylation, although not by the equivalent of 20 kDa (Figure S4). Whether the 95 kDa band reported by Wolter *et al.* [19] is a modified form of Gas2l3 is, therefore, unclear.

The third mode is protein localization: Gas2l3 localization in proliferating cells is dynamic. During interphase, the protein is associated with both the microtubule and the Actin-filament networks. During cell division, Gas2l3 shifts from the mitotic spindles to the midzone microtubules, and later to the midbody. At these sites, structural, regulatory, and motor proteins are assembled to regulate cytokinesis progression and mitotic exit [10].

### Gas2l3 is a Constriction Site-associated Protein whose Misregulation Interferes with Cell Abscission

Cell abscission occurs at the constriction sites. Some of the proteins involved include Spastin and the ESCRT-III complex subunit CHMP4b, which regulate microtubule severing and membrane scission, respectively [7], [8], [9]. We show that Gas2l3 is specifically localized to the narrowest part of the microtubule bundles in the intercellular bridge concomitantly with CHAM4b and Spastin. As such, Gas2l3 is a novel constriction site-associated protein (Figure 4).

Cells overexpressing Gas2l3 undergo cytokinesis and exit mitosis: the daughter cells decondense their chromatin, reform nuclei, reattach to the surface, and advance through the cell cycle, yet they remain connected by a thin and often abnormally long and misshaped intercellular bridge. This phenotype is significantly more prominent in cells expressing the D-box mutant derivate of Gas2l3, suggesting that it is not simply the result of saturating the APC/C^Cdh1^ machinery by the overexpressed Gas2l3, but is specific to the activity of Gas2l3. More importantly, in view of these findings, it is now easy to speculate that timely expression of Gas2l3, as controlled by APC/C^Cdh1^-mediated proteolysis, plays a role in finalizing cell division (Figure 5).

Abnormal intercellular bridges are also formed in response to Securin overexpression or stabilization (Securin, in concert with Separin, regulates sister-chromatid separation [29]). In this particular case, thin chromatin bridges connecting the two daughter cells are formed [30]. We were unable to detect chromatin in the intercellular bridges of Gas2l3 overexpressing cells, suggesting that the sister chromatids underwent full separation. It therefore seems that while the abnormal bridges induced by non-degradable Securin are an epiphenomenon of incomplete chromatid separation, high levels of Gas2l3 interfere specifically with cell abscission. Morphologically, however, the outcomes appear misleadingly similar.

The suggested role of Gas2l3 in cell abscission is in perfect agreement with the protein’s localization at the constriction sites, and it is plausible that Gas2l3 acts in concert with other constriction site-associated proteins, regulating microtubule severing and membrane scission. At present, we do not understand how Gas2l3 regulates cell abscission. There is no indication that the downregulation of Gas2l3 interferes specifically with cell abscission [19]; however, high levels of Gas2l3, and more so of its stable variant, certainly do so (Figure 5). In view of this observation, it is easy to speculate that Gas2l3 inhibits cell abscission, and that its destruction is required for properly terminating the cell division process. This notion is particularly compelling in view of Bastos and Barr’s elegant work which suggests that Plk1 negatively regulates cell abscission, and that its degradation by the APC/C^Cdh1^ targets Cep55 to the midbody and promotes cell abscission [6].

Alternatively, but not in a mutually exclusive manner, one can speculate that a protein that binds Actin and/or Tubulin may itself act as a scaffold protein [31], as shown for the adenomatous polyposis coli (APC) protein [32] and for spectraplakins [33]. Scaffold proteins are tethering agents with a biphasic mode of action (the “biphasic effect”); that is, too-low or too-high protein concentrations are suboptimal but an optimal outcome is achieved at an intermediate concentration (see reference [31] for an excellent explanation of the biphasic effect). Although the crystal structure of Gas2l3 is still a work in progress, one can speculate that this protein, be its role regulatory or structural, may function as a tethering agent that brings proteins, as well as membrane components, to the constriction sites to induce cell abscission. In such a scenario, the level of Gas2l3 at the mitotic exit must be perfectly balanced for cell abscission to be properly executed.

## Materials and Methods

### Plasmids

Human GAS2l3 cloned into the pCMV-SPORT6 vector was purchased from the ATCC (ATCC clone # 7192847). In all D-box mutant (DM) variants, Arg 1 and Leu 4 of each D-box were substituted with Gly and Val using QuikChange site-directed mutagenesis (Stratagene). Emi1 C-terminus (amino acids 299–447) cloned into pGEX-4T-3 was a gift from Dr. Tao Wu [22], and pCS2-hCdh1 was a gift from Dr. Andrea Ballabeni. The cloning of pET28-UbcH10, pET28-UbcH10^C114S^ (UbcH10^DN^), pET28-hSecurin, pCS2-hSecurin, and pCS2-hGeminin, was described in [21]; pCS2-Tome-1 was described in [24]. The expression vectors FLAG-tagged human CHAM4b and Myc-tagged human Spastin were kindly provided by Dr. Natalie Elia.

The sequences of human GAS2L3 and its D-box derivate GAS2L3-DM4 were amplified with primers flanked by XhoI (forward) and BamHI (reverse) sites and cloned into EGFP-N1 and EGFP-C1 vectors (Clontech). The EGFP-fused GAS2L3 and GAS2L3-DM4 constructs were amplified with primers flanked by Fse1 (forward) and Asc1 (reverse) sites and cloned into the pCS2 vector for *in vitro* and *in vivo* expression. In addition, GAS2L3 and GAS2L3-DM4 were amplified from the pCMV-SPORT6-GAS2L3 (ATCC) and pCMV-SPORT6-GAS2L3-DM4, respectively, with primers flanked by Fse1 (forward) and AscI (reverse) to generate the pCS2-hGAS2L3 and pCS2-hGAS2L3-DM4 plasmids. To generate a non-degradable (D-box mutant) derivate of human Cyclin B1, Arg 42 and Leu 45 were substituted with Gly and Val, respectively, using QuikChange site-directed mutagenesis. The mutant gene was then cloned into pCS2 vector in-frame with a 5′ 6xHis-tag. The vectors pEGFP-N1 and pCS2 were also used. All plasmids were sequenced.

### Tissue Culture, Cell Synchronization, and Transfection

HeLa, HeLa S3 (S3), and HEK293T (293) cells were maintained in DMEM (Gibco) supplemented with 10% fetal bovine serum (Gibco), penicillin, and streptomycin (Gibco) at 37°C, 5% CO_2_. For G1 extract preparation, S3 cells were grown in suspension (1 L spinner flask, 85 rpm) until population reached a density of approximately 5 × 10^5^ cells/ml. The cells were then incubated with 2 mM thymidine (Sigma-Aldrich) for 20–22 hrs, washed and released into fresh media for 3–3.5 hrs, and blocked again by incubating with 50 ng/ml nocodazole (Sigma-Aldrich) for 12 hrs. Cells were then washed twice with fresh, warm media, released for 3 hrs, and then harvested for generating G1 (APC/C^Cdh1^-active) extracts. Late-mitotic (APC/C^Cdc20^-active) extracts were prepared as follows: 293 cells were transiently transected (using TransIT®-293, Mirus Bio LLC) with His-tagged non-degradable human Cyclin B1. Because transfection yields exceeded 90% efficiency, nearly all cells were arrested in late mitosis 24 hrs post-transfection [31] and were therefore harvested directly from the plate without any enrichment. Synchronization was validated using FACSCalibur flow cytometer (BD) after propidium-iodide staining of ethanol-fixed cells (Figure S5). We used metafectene® (Biontex) as the transfection reagent. MG132 (Sigma-Aldrich) was added at a final concentration of 20 µM to block proteasome activity. RO-3306 (ALEXIS Biochemicals) and roscovitine (Sigma-Aldrich) were used at concentrations of 0.1 mM and 1 mM, respectively.

### Extract Preparation and Degradation Assays

Extracts of S3 and 293 cells were prepared as follows: synchronized cells were harvested, washed with PBS, lysed in swelling buffer (20 mM HEPES, pH 7.7, 2 mM MgCl_2_, 5 mM KCl, 1 mM DTT, EDTA-free protease inhibitor cocktail), supplemented with energy-regeneration mix (1 mM ATP, 7.5 mM creatine phosphate, 70 µg/ml creatine phosphokinase, 0.1 mM EGTA), and homogenized by freeze–thawing and passage through 20 G and 26 G needles successively. Extracts were cleared by subsequent centrifugations (5 min at 2,700×g; 45 min at 18,000×g) and stored at −80°C.

Degradation assays were performed using 20 µl cell extracts supplemented with 1 µl of 20X energy-regeneration mix, 1 µl of 10 mg/ml Ubiquitin (Boston Biochem), 1 µl of 10 mg/ml recombinant UbcH10 or UbcH10^DN^, 5 µL of either 1 mg/ml Emi1 C-terminus fragment, 3.5 mg/ml Securin, or PBS, and 1–2 µl radiolabeled IVT product expressed in reticulocytes (Promega) with ^35^S-labeled methionine (PerkinElmer). Samples were incubated at 30°C. Aliquots were taken every 20 or 40 min, removed into sample buffer, and snap-frozen on dry ice. Samples were resolved by SDS–PAGE and visualized by autoradiography.

To obtain interphase *X. laevis* egg extracts, eggs from HCG-injected frogs were collected and washed in 1X MMR. Eggs were dejellied with 2% cysteine, and activated by 1 µg/ml calcium ionophore (Sigma-Aldrich), and then washed successively in XB buffer and XB buffer with protease inhibitor mix (Roche), all at 18°C. Forty-five minutes post-activation, eggs were packed (1 min at 157×g) in an Ultraclear centrifuge tube (Beckman Coulter, Inc.) supplemented with cytochalasin B (Sigma-Aldrich), and crushed at 15680×g for 10 min. The tube was pierced just above the yolk pellet, and the cytoplasmic layer was collected by gravity for a second clarifying spin (10 min at 15680×g). The following reagents were then added to final concentrations of 1X energy-regeneration mix: 10 µg/ml cytochalasin B, 100 µg/ml cycloheximide (Sigma-Aldrich), and 4% glycerol. Extracts were stored at −80°C. Protein degradations were assayed in interphase egg extracts in the presence or absence of 1 nM recombinant Cdh1, or in extracts that were driven into mitosis by preincubation with 10 µg/ml Δ90 cyclin B1 for 40 min at room temperature. In both cases, 1 µl substrate was mixed with 15 µl extracts, 1 µl of 20X energy-regeneration mix, and 1 µl of 10 mg/ml Ubiquitin. Reactions were incubated at room temperature for a total of 2 hrs. Aliquots were taken every 30 min and processed, as described (see reference [27] for more details).

### Immunofluorescence and Microscopy

HeLa cells on coverslips (1.5) were fixed in 4% paraformaldehyde ([PFA] Sigma-Aldrich) for 20 min at room temperature (RT), blocked with 20% fetal bovine serum with 0.1% Triton X-100 in phosphate-buffered saline for 1 hr, and then incubated overnight with primary antibodies at 4°C or, alternatively, for 20–60 min at RT. After washing, coverslips were incubated with secondary antibodies. The DNA was visualized with 5 µg/ml DAPI (Sigma-Aldrich), and the coverslips were mounted on slides with Immu-Mount mounting solution (Thermo Shandon Limited) and sealed. Staining was analyzed using the AxioImager.Z1 upright and AxioObserver.M1 inverted fluorescence microscopes (Carl Zeiss, Inc.) equipped with 100X oil immersion lens objectives (NA 1.4). AxioVision Rel 4.8 (Carl Zeiss, Inc.) and ImageJ (NIH) software were used for image processing.

### Antibodies

The following primary antibodies were used for Western blotting: rabbit polyclonal anti-hGas2l3 (serum, custom-made by Covance), mouse monoclonal anti-αTubulin (DSHB, 12G10), and mouse monoclonal anti-β Actin (DSHB, JLA20). HRP-conjugated secondary antibodies were purchased from Jackson ImmunoResearch Inc. The following primary antibodies were used for IF: rabbit polyclonal anti-hGas2l3 (serum, custom-made by Covance), mouse monoclonal and rabbit polyclonal anti-Aurora B (Abcam, ab3609, ab2254), rabbit polyclonal MKLP1 (Gene Tex, GTX30315), mouse monoclonal anti-αTubulin (Abcam, ab7291), mouse monoclonal anti-FLAG® M2 (Sigma-Aldrich, F3165), and mouse monoclonal anti-Myc (DSHB, 9E10). Secondary antibodies used for IF include: Alexa Fluor 488-, 594-, 647-labeled donkey, anti-mouse, rabbit, or goat (Molecular Probes), A11029, A11008, A21201, A31571, A31573, and A21447), and Alexa Fluor 555-labeled goat anti-rabbit (Molecular Probes, A21429).

## Supporting Information

Figure S1 **Anti-hGas2l3 rabbit serum detects Gas2l3-EGFP at the constriction sites.** (A) HeLa cells were transfected with Gas2l3-EGFP and fixed with 4% PFA after 32 hrs. The fixed cells were immunolabeled with anti-hGas2l3 rabbit serum and Alexa Fluor 555 goat anti-rabbit secondary antibodies (Invitrogen). For imaging, we used the AxioImager.Z1 upright fluorescence microscope (Carl Zeiss, Inc.) equipped with 100X oil DIC immersion lens objectives (NA 1.4). (B) Fluorescence intensities of Gas2l3-EGFP and Gas2l3 at the midbody were quantified by linescan (ImageJ).
(PDF)

Figure S2 **siRNA experiments confirm Gas2l3 localization at the constriction sites and the specificity of anti-hGas2l3 serum.** (A) Real-time qPCR analysis of hGAS2L3 expression (relative quantification [RQ], normalized to hHPRT1 mRNA levels [in triplicates]) in HeLa cells treated with either hGAS2L3 siRNA (Sigma-Aldrich; EHU130291 Hs01 00152121_AS) or scrambled siRNA ([siControl] Sigma-Aldrich; SIC001). We used DharmaFECT (Thermo Scientific, D-001630-02), as transfection reagent, following the manufacturer’s protocol. Cells were harvested for RNA extraction 40 h post-transfection. Primers used for real-time qPCR: HPRT1-RT-F: CGTGATTAGTGATGATGAACCAG; HPRT1-RT-R: CGAGCAAGACGTTCAGTCCT; hGAS2L3-RT-F: GCTGTCGGCATGAAGAGC; hGAS2L3-RT-R: AATCGATGAGAACAA CTACAAGGA. (B) HeLa cells were cotransfected (DharmaFECT) with Gas2l3-EGFP or empty pCS2 vector (-), and with either a negative control (siControl) or GAS2L3 siRNAs. Forty hrs post-transfection, cells were harvested for Western blot analysis with anti-GFP (Santa Cruz) and anti-Actin ([loading control], Sigma-Aldrich, A3853). (C) HeLa cells were cotransfected with Gas2l3DM4-EGFP and with either a negative control (siControl) or GAS2L3 siRNAs. We used DharmaFECT as a transfection reagent. Transfection mix included siGLO to detect siRNA positive cells. Thirty-six hrs post-transfection, cells were fixed (4% PFA), stained with DAPI, and imaged (100X DIC oil lens). White arrows indicate Gas2l3 at the constriction sites. (D) HeLa cells were transfected (DharmaFECT) with either hGAS2L3 siRNA or scrambled siRNA (siControl). Transfection mix included siGLO red transfection indicator to detect siRNA positive cells. Thirty-six hrs post-transfection, cells were fixed (4% PFA), immunolabeled with anti-hGas2l3 rabbit serum and Alexa Fluor 555 goat anti-rabbit secondary antibodies (Invitrogen), and stained with DAPI. For imaging, we used the AxioImager.Z1 upright fluorescence microscope (Carl Zeiss, Inc.) equipped with 100X oil DIC immersion lens objectives (NA 1.4).
(PDF)

Figure S3 **The localization of Gas2l3 at the constriction sites is unaffected by the D-box.** HeLa cells were transfected with EGFP-Gas2l3 (A) or the D-box–mutant derivate of Gas2l3-EGFP (Gas2l3-DM4-EGFP) (B). Cells were fixed (4% PFA) 32 hrs post-transfection and immunolabeled with anti-Aurora B antibodies (Abcam, ab2254) and Alexa Fluor 555 goat anti-rabbit secondary antibodies (Invitrogen). For imaging, we used the AxioImager.Z1 upright fluorescence microscope (Carl Zeiss, Inc.) equipped with 100X oil immersion lens objectives (NA 1.4).
(PDF)

Figure S4 **Gas2l3 mobility in SDS-PAGE is hardly shifted by mitotic phosphorylation.** Radiolabeled Gas2l3 IVT product was incubated in 293 late mitotic cell extracts (see main text) for 15 min in the presence (+) of the Cdk1 inhibitor RO-3360 or with DMSO ([-] control). In addition, after 15 min of incubation, extracts supplemented with either RO-3360 or DMSO were mixed (+/−). Samples were assayed by SDS-PAGE and autoradiography. Radiolabeled EGFP-Gas2l3 is being used as a 104 kDa marker, 29 kDa higher than the calculated Gas2l3 molecular weight (MW) of 75 kDa.
(PDF)

Figure S5 **HEK293 cells expressing non-degradable full-length Cyclin B1 arrest in mitosis.** HEK293 cells were transfected with non-degradable full-length Cyclin B1 (ndCycB1). Thirty hrs post-transfection, cells were harvested for FACS analysis of their DNA content (PI staining). In addition, asynchronous (US) and nocodazole-arrested (Noc) 293 cells were harvested for FACS analysis. All samples were fixed (70% EtOH), stained with PI, and assayed using FACSCalibur (BD) and ModFit LT™ software.
(PDF)
