# Supplementary material for: Gas2l3, a Novel Constriction Site-Associated Protein Whose Regulation Is Mediated by the APC/CCdh1 Complex
Source: PLoS One. 2013 Feb 28;8(2):e57532. doi: 10.1371/journal.pone.0057532 (PMC3585356; doi:10.1371/journal.pone.0057532)

Figure S1. Anti-hGas2l3 rabbit serum detects Gas2l3-EGFP at the constriction sites

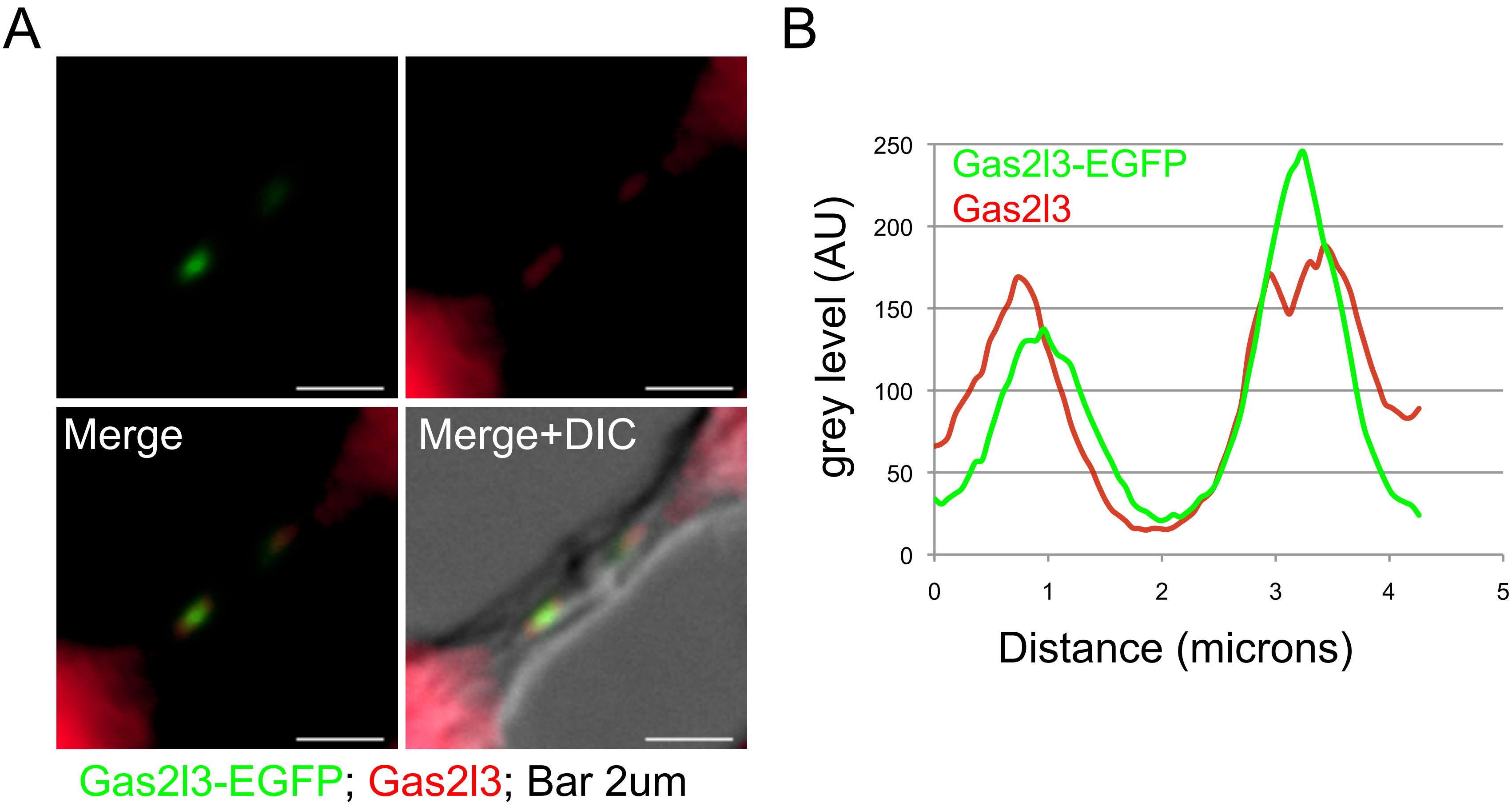

Supplement: Figure S1 — Anti-hGas2l3 rabbit serum detects Gas2l3-EGFP at the constriction sites. (A) HeLa cells were transfected with Gas2l3-EGFP and fixed with 4% PFA after 32 hrs. The fixed cells were immunolabeled with anti-hGas2l3 rabbit serum and Alexa Fluor 555 goat anti-rabbit secondary antibodies (Invitrogen). For imaging, we used the AxioImager.Z1 upright fluorescence microscope (Carl Zeiss, Inc.) equipped with 100X oil DIC immersion lens objectives (NA 1.4). (B) Fluorescence intensities of Gas2l3-EGFP and Gas2l3 at the midbody were quantified by linescan (ImageJ). (PDF) [file pone.0057532.s001.pdf]
