# Supplementary material for: Gas2l3, a Novel Constriction Site-Associated Protein Whose Regulation Is Mediated by the APC/CCdh1 Complex
Source: PLoS One. 2013 Feb 28;8(2):e57532. doi: 10.1371/journal.pone.0057532 (PMC3585356; doi:10.1371/journal.pone.0057532)

Figure S2. siRNA experiments confirm Gas2l3 localization at the constriction sites and the specificity of the anti-hGas2l3 serum

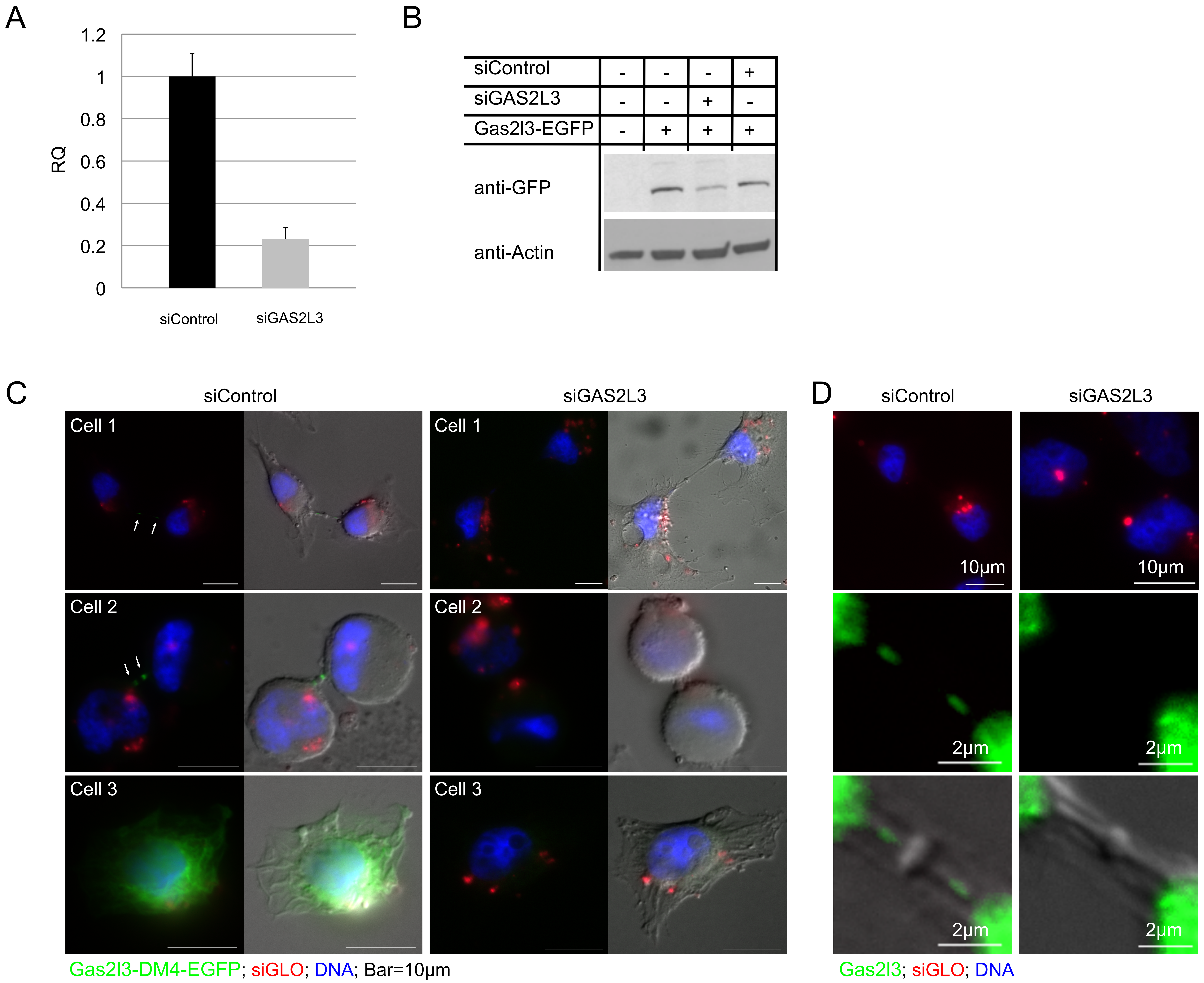

Supplement: Figure S2 — siRNA experiments confirm Gas2l3 localization at the constriction sites and the specificity of anti-hGas2l3 serum. (A) Real-time qPCR analysis of hGAS2L3 expression (relative quantification [RQ], normalized to hHPRT1 mRNA levels [in triplicates]) in HeLa cells treated with either hGAS2L3 siRNA (Sigma-Aldrich; EHU130291 Hs01 00152121_AS) or scrambled siRNA ([siControl] Sigma-Aldrich; SIC001). We used DharmaFECT (Thermo Scientific, D-001630-02), as transfection reagent, following the manufacturer’s protocol. Cells were harvested for RNA extraction 40 h post-transfection. Primers used for real-time qPCR: HPRT1-RT-F: CGTGATTAGTGATGATGAACCAG; HPRT1-RT-R: CGAGCAAGACGTTCAGTCCT; hGAS2L3-RT-F: GCTGTCGGCATGAAGAGC; hGAS2L3-RT-R: AATCGATGAGAACAA CTACAAGGA. (B) HeLa cells were cotransfected (DharmaFECT) with Gas2l3-EGFP or empty pCS2 vector (-), and with either a negative control (siControl) or GAS2L3 siRNAs. Forty hrs post-transfection, cells were harvested for Western blot analysis with anti-GFP (Santa Cruz) and anti-Actin ([loading control], Sigma-Aldrich, A3853). (C) HeLa cells were cotransfected with Gas2l3DM4-EGFP and with either a negative control (siControl) or GAS2L3 siRNAs. We used DharmaFECT as a transfection reagent. Transfection mix included siGLO to detect siRNA positive cells. Thirty-six hrs post-transfection, cells were fixed (4% PFA), stained with DAPI, and imaged (100X DIC oil lens). White arrows indicate Gas2l3 at the constriction sites. (D) HeLa cells were transfected (DharmaFECT) with either hGAS2L3 siRNA or scrambled siRNA (siControl). Transfection mix included siGLO red transfection indicator to detect siRNA positive cells. Thirty-six hrs post-transfection, cells were fixed (4% PFA), immunolabeled with anti-hGas2l3 rabbit serum and Alexa Fluor 555 goat anti-rabbit secondary antibodies (Invitrogen), and stained with DAPI. For imaging, we used the AxioImager.Z1 upright fluorescence microscope (Carl Zeiss, Inc.) equipped with 100X oil DIC immersion lens objec [file pone.0057532.s002.pdf]
