# Supplementary figures and images for: Gas2l3, a Novel Constriction Site-Associated Protein Whose Regulation Is Mediated by the APC/CCdh1 Complex
Source: PLoS One. 2013 Feb 28;8(2):e57532. doi: 10.1371/journal.pone.0057532 (PMC3585356; doi:10.1371/journal.pone.0057532)

**Figure S3.** The localization of Gas2l3 at the constriction sites is unaffected by the D-box

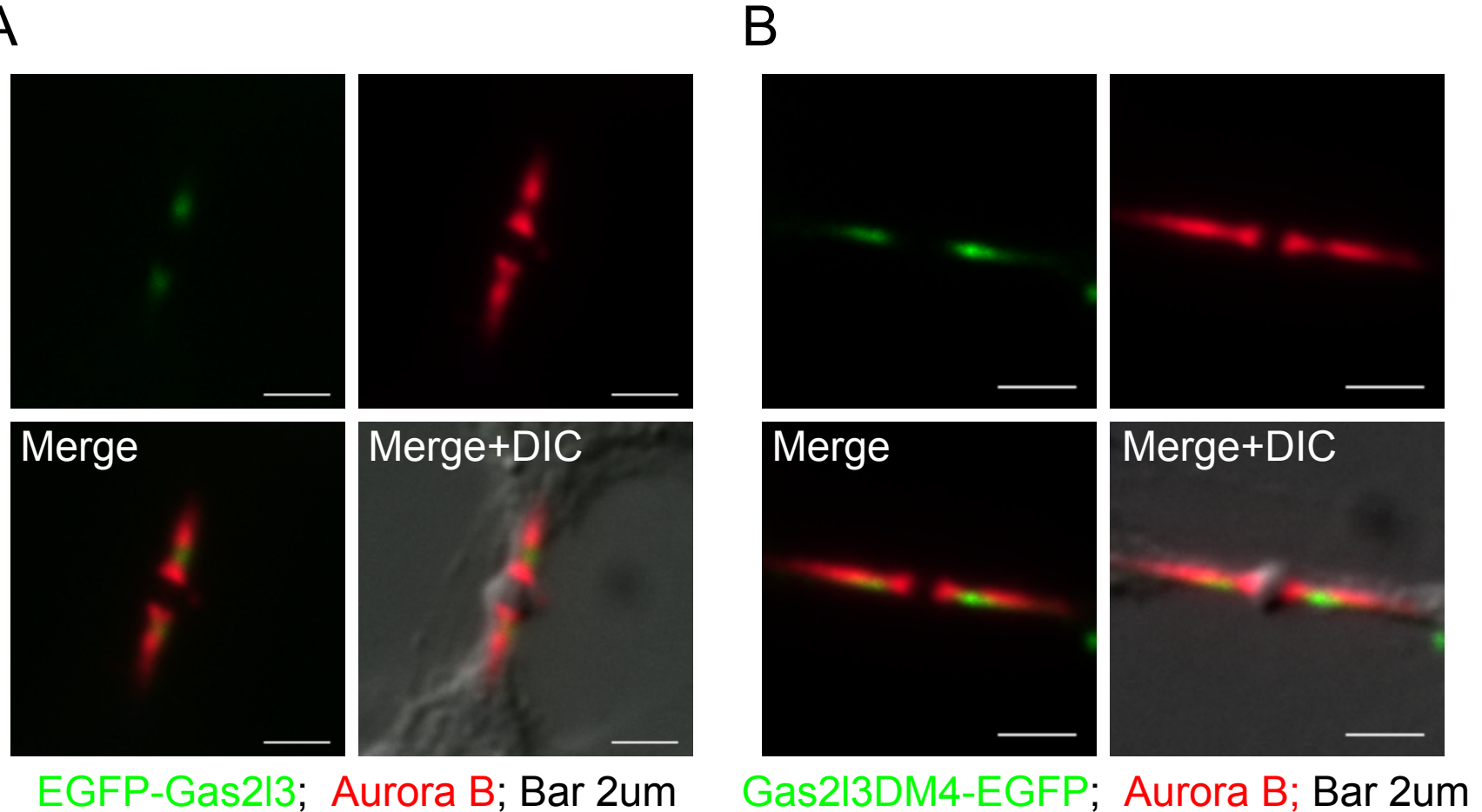

Supplement: Figure S3 — The localization of Gas2l3 at the constriction sites is unaffected by the D-box. HeLa cells were transfected with EGFP-Gas2l3 (A) or the D-box–mutant derivate of Gas2l3-EGFP (Gas2l3-DM4-EGFP) (B). Cells were fixed (4% PFA) 32 hrs post-transfection and immunolabeled with anti-Aurora B antibodies (Abcam, ab2254) and Alexa Fluor 555 goat anti-rabbit secondary antibodies (Invitrogen). For imaging, we used the AxioImager.Z1 upright fluorescence microscope (Carl Zeiss, Inc.) equipped with 100X oil immersion lens objectives (NA 1.4). (PDF) [file pone.0057532.s003.pdf]

**Figure S4.** Gas2l3 mobility in SDS-PAGE is hardly shifted by mitotic phosphorylation

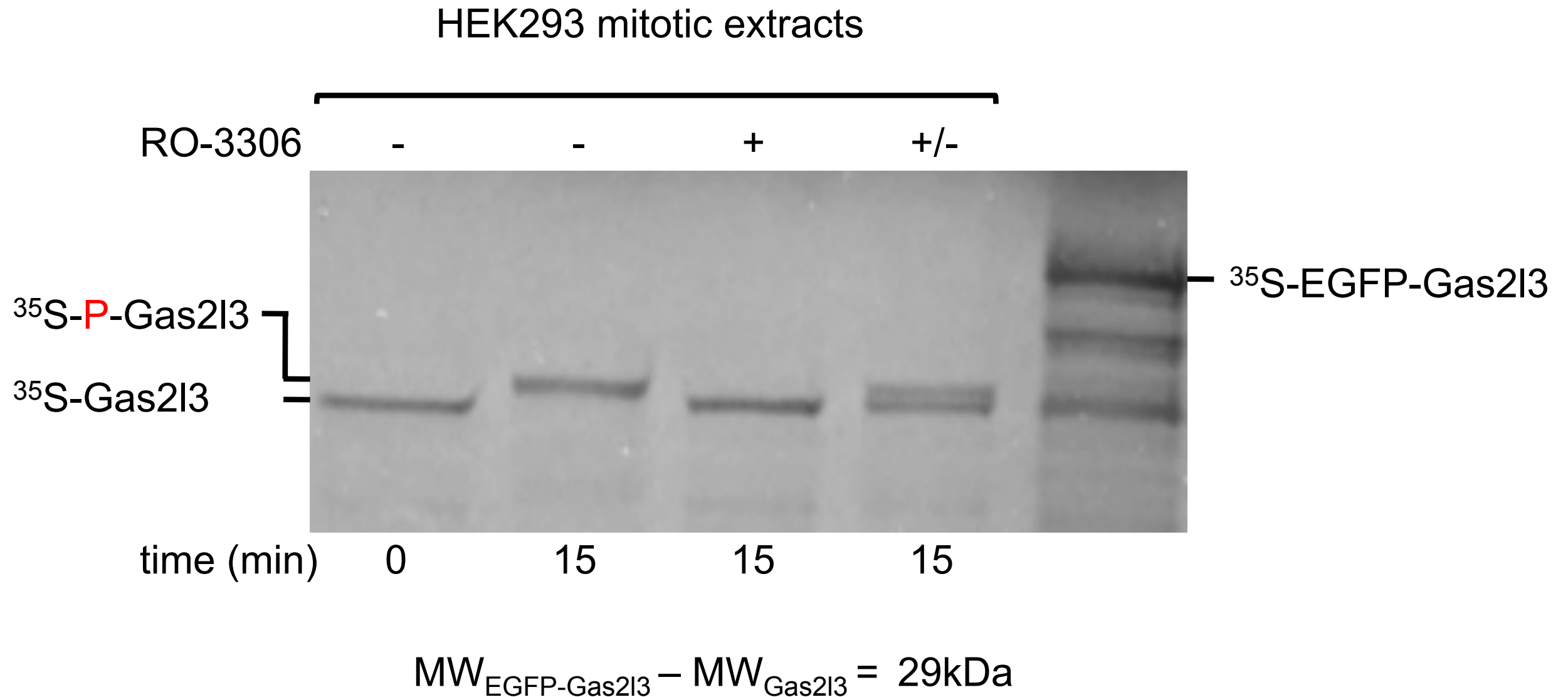

Supplement: Figure S4 — Gas2l3 mobility in SDS-PAGE is hardly shifted by mitotic phosphorylation. Radiolabeled Gas2l3 IVT product was incubated in 293 late mitotic cell extracts (see main text) for 15 min in the presence (+) of the Cdk1 inhibitor RO-3360 or with DMSO ([-] control). In addition, after 15 min of incubation, extracts supplemented with either RO-3360 or DMSO were mixed (+/−). Samples were assayed by SDS-PAGE and autoradiography. Radiolabeled EGFP-Gas2l3 is being used as a 104 kDa marker, 29 kDa higher than the calculated Gas2l3 molecular weight (MW) of 75 kDa. (PDF) [file pone.0057532.s004.pdf]

**Figure S5.** HEK293 cells expressing non-degradable full-length Cyclin B1 arrest in mitosis

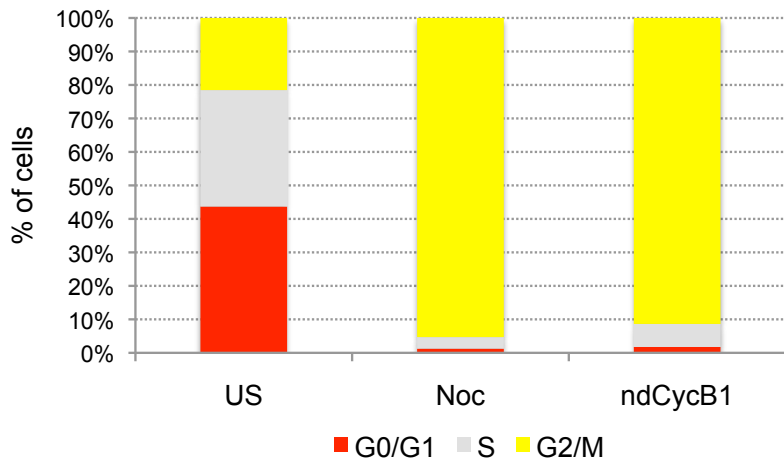

Supplement: Figure S5 — HEK293 cells expressing non-degradable full-length Cyclin B1 arrest in mitosis. HEK293 cells were transfected with non-degradable full-length Cyclin B1 (ndCycB1). Thirty hrs post-transfection, cells were harvested for FACS analysis of their DNA content (PI staining). In addition, asynchronous (US) and nocodazole-arrested (Noc) 293 cells were harvested for FACS analysis. All samples were fixed (70% EtOH), stained with PI, and assayed using FACSCalibur (BD) and ModFit LT™ software. (PDF) [file pone.0057532.s005.pdf]
